# Occurrence and Morphometric Characterization of *Cysticercus tenuicollis* in Sheep From Minas Gerais, Brazil

**DOI:** 10.1155/vmi/8489002

**Published:** 2026-07-07

**Authors:** Cintia Aparecida de Jesus Pereira, Valdir de Miranda Brito, Ana Luisa Gomes, Raphael Meira Becattini, Gabriel Luiz Vieira Mendes, Samuel Tadeu Rocha, Weder Gomes Oliveira, Guilherme Henrique Rosemberg Silva, Sabrina Aparecida Batista Maia de Oliveira, Walter dos Santos Lima

**Affiliations:** ^1^ Departamento de Parasitologia, Universidade Federal de Minas Gerais, Belo Horizonte, Minas Gerais, Brazil, ufmg.br

**Keywords:** canids, *Cysticercus tenuicollis*, environment, sheep farming

## Abstract

In livestock production, the health of slaughtered animals is crucial to ensuring food safety. In this context, animal cysticercosis poses serious problems within production systems. Cysticercosis caused by *Cysticercus tenuicollis* (larval stage of *Taenia hydatigena*) is common in pasture‐raised ruminants. Although this helminthiasis is of low pathogenicity for production animals, it is difficult to diagnose during inspection procedures. Animals become infected by ingesting eggs present in water and/or feed contaminated with feces from domestic or wild canids. In this study, viscera from Dorper/Santa Inês crossbred sheep raised under a semiextensive system were evaluated in situ during four slaughter events in a slaughterhouse in the metropolitan region of Minas Gerais. Among 700 sheep slaughtered, 113 cysticerci were recovered and stored at 4°C for 3 h. They were then subjected to evagination using 1% hydrochloric acid/pepsin at 37°C for 10 min. Stereomicroscopy revealed cysticerci measuring 0.6 cm in length and 0.2 cm in width when invaginated and ranging from 2 to 7 cm in length and 1.5 to 8 cm in width when viable and evaginated forms. A total of 25.7% of parasites were found in the liver and 74.3% in the omentum, mesentery, and associated tissues. Microscopy also allowed the observation of rostellar hook morphology regarding number, width, and total length of large and small hooks. This study reports the first occurrence of ovine cysticercosis by *C. tenuicollis* in Minas Gerais, Brazil.

## 1. Introduction

Endoparasitoses are major factors affecting livestock production, reducing farmers’ profits, and limiting the economic productivity of ruminant herds in Brazil and worldwide [1]. In Brazil, ovine cysticercosis by *Cysticercus tenuicollis* has been reported in Pernambuco [2], Piauí [3], Rio Grande do Sul [4], Paraná [5], and Paraíba [6]. Although considered a foodborne disease (FBD), it remains understudied. The definitive hosts, domestic and wild canids, harbor parasites in the small intestine that may reach up to 5 m in length. Eggs are released into the environment via feces, and intermediate hosts become infected after ingestion. Once ingested, the eggs are digested by gastric and intestinal secretions, releasing oncospheres that penetrate the intestinal wall and migrate to viscera such as the omentum and mesentery, where larval development is completed [7].

Cattle, sheep, and goats are considered the main intermediate hosts for the larval stage of this parasitosis [7]. However, swine and horses may also be affected [6, 8]. Four to five weeks after ingestion of eggs, immature forms reach an average of 6–8 cm in diameter [7]. Definitive hosts become infected after ingesting cysticerci present in the viscera of infected production animals [9–11].

When viable, cysticerci are translucent or slightly opaque cystic structures filled with transparent fluid, within which the scolex can be observed, whereas calcified cysts are firm [12].

## 2. Materials and Methods

### 2.1. Sample Collection and Identification of Cysticerci

Viscera from slaughtered male sheep, up to 12 months of age, crossbred Dorper/Santa Inês, and originating from the central region of Minas Gerais, were examined at slaughter. Cysticerci were sampled through in situ evaluation during four slaughter events. Hooks were dissected from 10 randomly selected, well‐developed cysticerci. This sample size was considered adequate, as all infected animals originated from a single herd shared the same geographical location and were subjected to uniform management and environmental conditions. Such epidemiological homogeneity supports the assumption of low intragroup variability among the parasites​ [13]. Morphological and morphometric identification was performed on 10 large hooks and 10 small hooks.

Cysticerci found in the liver, omentum, mesentery, and tissues adjacent to the intestine were fully removed, packed, and transported to the laboratory on ice (4°C) in insulated boxes. The material was washed in 0.85% saline solution and maintained refrigerated at 4°C in RPMI 1640 medium supplemented with 10% fetal bovine serum. It was then subjected to chemical digestion using 1% hydrochloric acid/pepsin at 37°C for 10 min [14]. After evagination, parasites were compressed between glass slides and fixed in 4% formalin.

The Differential Interference Contrast (DIC) technique was employed to visualize rostellar structures of *Cysticercus tenuicollis*, providing high‐contrast, pseudo‐relief images without staining [15]. Laser scanning confocal microscopy with spectral imaging was used for detailed analysis. Imaging was performed using a Zeiss LSM 880 confocal microscope equipped with a 32‐channel GaAsP spectral detector, enabling high sensitivity and improved signal‐to‐noise ratio. Optical sectioning was achieved using a pinhole set to 1 Airy Unit, allowing the acquisition of high‐resolution images and 3D reconstruction [16–18].

Spectral imaging was applied to obtain detailed information on emission profiles. In this approach, a spectral scan is performed at each pixel, generating a spectral stack that records the distribution of emission wavelengths across the entire image. To distinguish intrinsic autofluorescence of *Cysticercus tenuicollis* from the signals of fluorescent markers, the Linear Unmixing algorithm integrated into the ZEN Black software (Zeiss) was used. Images were captured using a Zeiss LSM 880 microscope with an EC Plan‐Neofluar 20x/0.50 NA objective. For DIC imaging, a 488‐nm laser was used with Scan Zoom 0.64×, pixel time 0.76 µs, T‐PMT gain 427 V, offset 0, and 8‐bit depth. Spectral imaging employed 405‐, 488‐, 543‐, and 633‐nm excitation lasers, with Lambda mode beam splitters (MBS 488/543/633 and MBS InVis 405). Acquisition parameters included Scan Zoom 0.64×, pixel time 1.24 µs, averaging 1, spectral range 410–694 nm, and resolution of 8.8 nm/channel. Detector gain was set to 712 V with offset 0. Linear Unmixing (ZEN Black) was applied to separate autofluorescence from fluorophore signals based on emission spectra.

Seven parameters were analyzed: total width (TW) and total length (TL) of cysticerci and rostellar hook morphology. Scolices were placed on glass slides, and pressure was applied to the coverslip to release the hooks, enabling detailed measurements: number of hooks per rostellum (NUH), total width of large hooks (TWLH), total width of small hooks (TWSH), total length of large hooks (TLLH), and total length of small hooks (TLSH).

## 3. Results

### 3.1. Presence of *Cysticercus tenuicollis* in Evaluated Samples

Viscera from 700 lambs were examined, and a total of 113 cysticerci were identified: 29 in the liver (25.7%) and 84 in the omentum, mesentery, and tissues adjacent to the intestine (74.3%). Liver lesions were observed, with 28 calcified cysticerci and only one viable cysticercus. In contrast, no calcified cysticerci were found in the mesentery, omentum, or adjacent tissues, where 84 viable cysticerci were identified (see Table 1).

**TABLE 1:** *Cysticercus tenuicollis* obtained from fragments of intestine, omenta, and livers *in loco* during the slaughter of 700 sheeps from the central region of Minas Gerais.

Number of sheeps slaughtered	Organs/tissues examined	*Cysticercus tenuicollis*		Prevalence (%)
		Viable	Calcified	
200	Liver	1	28	25.7
150	Omentum/mesentery/tissues adjacent to intestine	26	0	
170	Omentum/mesentery/tissues adjacent to intestine	28	0	74.3
180	Omentum/mesentery/tissues adjacent to intestine	30	0	

*Total*				
700		85	28	100

### 3.2. Morphological and Morphometric Identification of *Cysticercus tenuicollis*

**FIGURE 1:**
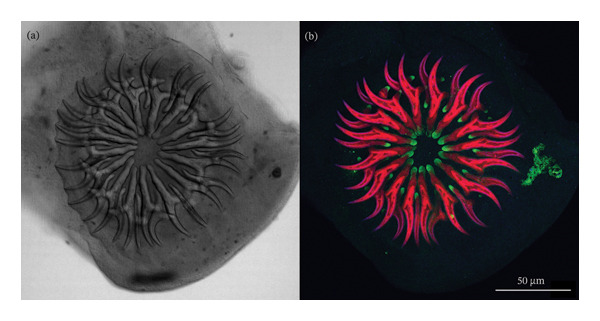
(a) DIC image and (b) confocal microscopy with spectral imaging of the rostellum of *Cysticercus tenuicollis*.

Morphological and morphometric characteristics of the scolex were used to characterize cysts found in the mesentery, omentum, liver, and tissues adjacent to the intestine. A double row of hooks was observed in the rostellum (see Figure 1).

**FIGURE 2:**
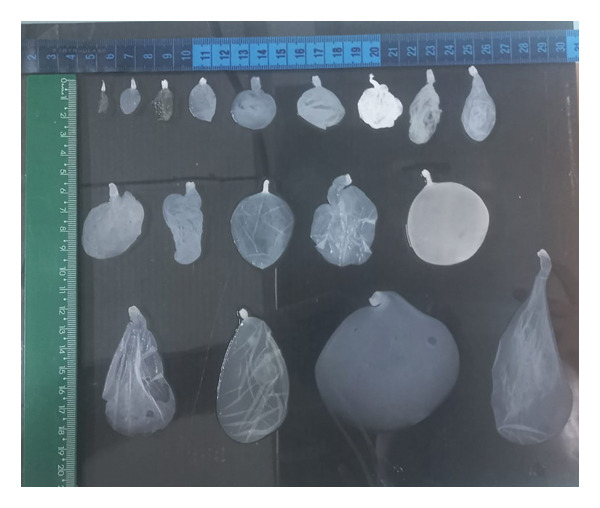
*Cysticercus tenuicollis*, larvae of *Taenia hydatigena*.

Viable cysts of *C. tenuicollis* are characterized by thin walls, with transparent or milky appearance. Chemically stimulated vcysticerci undergoing evagination showed heterogeneous sizes, ranging from 2 to 7 cm in length and 1.5 to 8 cm in width (Figure 2).

Morphometric analysis of the rostellum, as illustrated in Figure 1, was performed on five rostelli. The total NUH ranged from 32 to 34 (33.4 ± 0.89). The first row comprised 16‐17 large hooks (NULH) (16.8 ± 0.44), while the second row contained 16‐17 small hooks (NUSH) (16.6 ± 0.54). The mean TWLH ranged from 58.39 to 73.37 μm (65.38 ± 4.42 μm), and the mean TLLH ranged from 182.66 to 196.91 μm (189.75 ± 4.68 μm). The mean TWSH ranged from 50.84 to 65.46 μm (58.93 ± 4.16 μm), while the mean TLSH ranged from 130.72 to 141.36 μm (137.50 ± 3.25 μm) (Figure 3).

FIGURE 3Mean values of the hooks of the scolex of *Cysticercus tenuicollis*. (a) Small hooks. (b) Large hooks.

**Figure:**
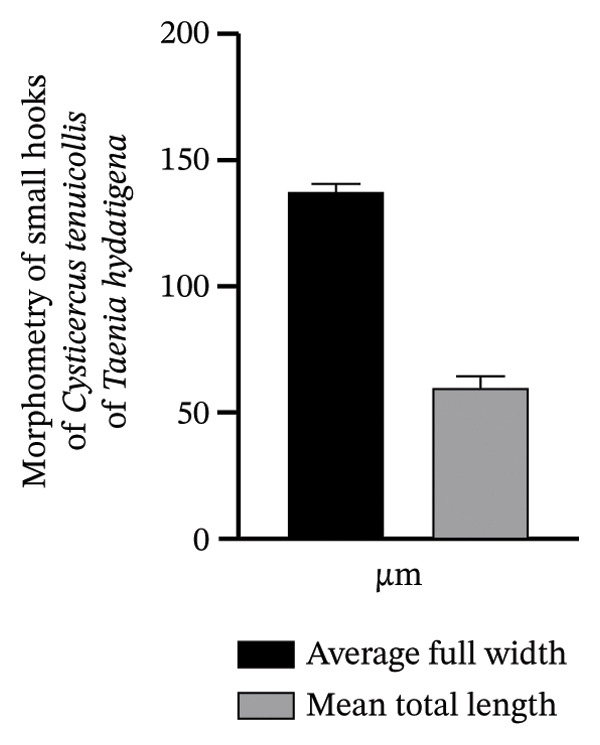
(a)

**Figure:**
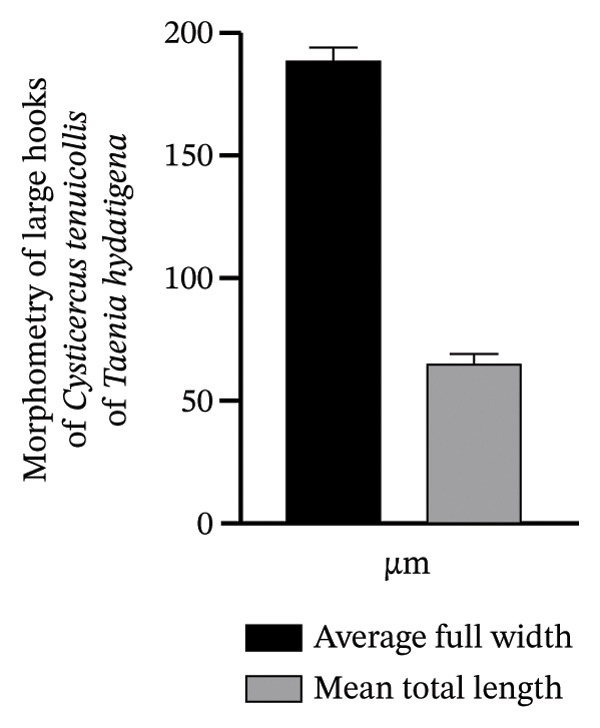
(b)

## 4. Discussion

Despite its low pathogenicity, *Cysticercus tenuicollis* is frequently detected in pasture‐raised animals, with a predominance in the omentum and liver. Its presence in the liver is of particular concern, as it can result in condemnation of the organ or even the entire carcass, as cysticercosis is subject to mandatory reporting in meat inspection systems. Therefore, differential diagnosis from *Taenia saginata* cysticercus (*Cysticercus bovis*), *T. ovis* (*C. ovis*), and, in some cases, hydatid cysts in the liver is essential for accurate diagnosis and for understanding the epidemiological dynamics of these infections, including those with zoonotic potential.

In this context, the work of Sweatman and Plummer is highly relevant, as it provides a detailed description of the development of *T. hydatigena* cysticerci and contributes to a better understanding of the parasite’s biology [19]. The hepatic fibrotic foci indicative of embryonic cysticerci first appeared in lambs with 7‐day infections, and macroscopic cysticerci (1–9 mm), most within hemorrhagic streaks, occurred at 10 days. Migration of cysticerci into the abdominal cavity from streaks and open pits in the liver capsule occurred in 18‐ to 25‐day infections and from the omentum in 25‐ and 30‐day infections. Cysticerci in the abdominal fluid were last observed at 43 days. Most cysticerci that remained within the liver parenchyma of lambs became entombed within caseous and fibrotic material and failed to complete their rostellar development, irrespective of the period of infection. Those located at other sites developed suckers and complete hooks between 34 and 53 days. Cysticerci within the parenchyma of a moose completed their development. Hyalinosis of some omental cysts in lambs occurred at 25 days, although the cysticerci remained viable until at least 53 days, when some became infiltrated with caseous material. A fibroma was associated with an omental cyst. Crystallized flakes were observed in teased hepatic haemorrhagic streaks and omental cysts in 4‐month infections, followed by cyst size regression. Adhesions and ribbons of fibrotic tissue, most commonly involving the liver and diaphragm or the omentum and body wall, were first observed in infections of 30 and 61 days, respectively. In infections longer than 53 days, the number of viable or degenerated cysticerci in lambs and swine at sites other than the liver parenchyma was proportional to the total number of cysts. The growth rate of viable cysticerci remained constant both before and after complete rostellar development.

The difficulty in recognizing larval stages of cestodes remains a major challenge and contributes to the persistence and spread of these helminthiases. Postmortem inspection of viscera in slaughterhouses was fundamental in this study, not only for the collection of epidemiological data but also for the morphometric and morphological identification of scolices and rostelli of *C. tenuicollis*.

In Brazil, economic losses associated with *T. hydatigena* cysticerci in sheep production occur primarily when cysticerci are detected in the liver, where they may be confused with those of *T. saginata*. When viable or calcified cysticerci are observed in the liver, carcasses may be removed from the production line, downgraded in value, or even condemned. Definitive differentiation relies on morphological confirmation through evagination of viable cysticerci, as demonstrated in Figure 2, which allows distinction between *Cysticercus tenuicollis* and *Cysticercus bovis*. In contrast, cysticerci located in the omentum, peritoneum, and mesentery are typically mature and well developed, which facilitates evagination. This process enables differentiation based on rostellar features and hook morphology, as demonstrated in this study (Figure 1).

Although molecular confirmation of the species was not performed, this study conducted a detailed characterization of hook morphology through individual dissection. The alternating arrangement of hooks in double rows, the number of large and small hooks, and their morphometric characteristics are consistent with findings reported in previous studies [8, 13, 20–23].

Our findings are consistent with earlier reports describing a high frequency of *T. hydatigena* cysticerci in the omentum, mesentery, and tissues adjacent to the intestine [24, 25].

It is also important to highlight the limitations of serological diagnosis in cases of cysticercosis, as cross‐reactions are common. This is due to the continuous exposure of pasture‐raised animals to multiple cestode species throughout their lifetime, which complicates serological interpretation.

This is the first report of *C. tenuicollis* in sheep in Minas Gerais. However, the true prevalence of this parasite may be underestimated, as cysticerci located in the liver can be misidentified as larval stages of other *Taenia* species. Furthermore, their frequent occurrence in the omentum and intestinal tissues complicates detection, since these viscera are often washed and diverted for by‐product processing (e.g., animal feed production) in slaughterhouses, where they are not subjected to the same rigorous inspection as red viscera such as the liver. These practices likely contribute to underreporting and reinforce the need for more systematic surveillance strategies.

In the present study, a significant number of *T. hydatigena* cysticerci were observed in sheep from a farm located in the Central mesoregion of Minas Gerais, in the municipality of Curvelo (18°52′25.7″S, 44°36′32.4″W). On this farm, animals are raised under a semiconfinement system; until weaning, lambs have free access to pasture and natural water sources. This production system, along with the presence of domestic and/or wild canids accessing farm areas, is similar to that observed in other regions of the state and across Brazil, suggesting that the prevalence observed here may reflect conditions in extensive and semiextensive systems nationwide. However, further studies are needed to confirm the existence of the biological cycle between domestic and/or wild canids and ruminants on farms, since canids play a key role in disseminating cestode eggs through feces, contaminating water sources and pastures.

Within the context of sheep farming in Minas Gerais, this report is particularly relevant, as this livestock sector is expanding across several mesoregions of the state. Minas Gerais comprises 853 municipalities over a territorial area of approximately 586,514 km^2^, divided into 12 mesoregions. Sheep farming for meat production and subsistence is present in the Northern region (89 municipalities), Jequitinhonha (51 municipalities), the Metropolitan Region of Belo Horizonte (34 municipalities), Vertentes and Zona da Mata (143 municipalities), and the South and Southwest of Minas Gerais (140 municipalities). This distribution underscores the need to expand this research with larger sample sizes per region and to adopt an integrative taxonomy approach combining morphological and molecular data. Such an approach will enable identification of parasite diversity across different production systems (extensive, semiintensive, and intensive) in Minas Gerais [26]. Epidemiological data on ovine cysticercosis caused by *T. hydatigena* in Brazil remain scarce, and even in traditionally important sheep‐producing regions such as Rio Grande do Sul, the number of animals evaluated has been limited [27].

## 5. Conclusion

This study provides the first report of ovine cysticercosis caused by *Cysticercus tenuicollis* in Minas Gerais, Brazil, thereby expanding the known distribution of *Taenia hydatigena* in the country. Although considered of low pathogenicity, the parasite was detected in a significant proportion of slaughtered sheep, particularly in the omentum and liver, where it may compromise organ integrity and lead to carcass condemnation. These findings reinforce the economic and sanitary relevance of this cestode infection in sheep farming.

The frequent occurrence of viable cysticerci in tissues not routinely subjected to thorough inspection highlights the risk of underreporting and indicates potential failures in the current surveillance system. The results also underscore the importance of accurate morphological and morphometric characterization for differential diagnosis, given the similarity of lesions with those produced by other *Taenia* spp. and hydatid cysts.

The presence of *T. hydatigena* in semiextensive production systems suggests active maintenance of both domestic and sylvatic transmission cycles, with contamination of pastures, water, and feed by canid feces representing a critical risk factor. Control strategies should include improved farm management, restriction of dog access to slaughter waste, and farmer education on the epidemiology of cestode infections.

Taken together, these findings emphasize the need for more comprehensive monitoring and stricter sanitary measures to reduce economic losses and strengthen food safety in sheep production.

## Funding

This work was carried out without financial support from external sources.

## Conflicts of Interest

The authors declare no conflicts of interest.

## Data Availability

The data that support the findings of this study are available on request from the corresponding author. The data are not publicly available due to privacy or ethical restrictions.
